# Swimming Using Surface Acoustic Waves

**DOI:** 10.1371/journal.pone.0042686

**Published:** 2013-02-19

**Authors:** Yannyk Bourquin, Jonathan M. Cooper

**Affiliations:** Division of Biomedical Engineering, University of Glasgow, Glasgow, United Kingdom; University of Manchester, United Kingdom

## Abstract

Microactuation of free standing objects in fluids is currently dominated by the rotary propeller, giving rise to a range of potential applications in the military, aeronautic and biomedical fields. Previously, surface acoustic waves (SAWs) have been shown to be of increasing interest in the field of microfluidics, where the refraction of a SAW into a drop of fluid creates a convective flow, a phenomenon generally known as SAW streaming. We now show how SAWs, generated at microelectronic devices, can be used as an efficient method of propulsion actuated by localised fluid streaming. The direction of the force arising from such streaming is optimal when the devices are maintained at the Rayleigh angle. The technique provides propulsion without any moving parts, and, due to the inherent design of the SAW transducer, enables simple control of the direction of travel.

## Introduction

Surface acoustic waves (SAWs) are commonly known to engineers as mechanical waves that can be induced at the interface of a transducer, often using the electrical stimulation of a piezoelectric substrate. In such a configuration, they find applications in the telecommunications industry, where the electro-mechanical coupling is used as an electronic filter or tuner. Recently, they have also been shown to be of increasing interest in the field of microfluidics, where the refraction of a SAW into a drop of fluid creates a convective flow, a phenomenon generally known as SAW streaming [1], [2]. In one example, the jetting of fluid from a sessile drop has been demonstrated, following the application of a SAW [3], [4]. Interestingly, although SAWs have been used to actuate a rotor by friction, to create a mechanical displacement, [5] their use as a direct mean for propulsion remains untested.

Recent interest in microactuation of free standing objects in fluids arises from their potential applications in the military, aeronautic and biomedical fields. For example, small exploratory aquatic vehicles may be used for exploration, whilst swimming microrobots have been proposed for minimally invasive surgery and endoscopy [6]. Such propulsion technologies generally use rotary propellers actuated using either electrostatic, [7] electromagnetic, [8] piezoelectric, [6] osmostic [9] or thermal [10] driving forces. In such cases the miniaturization of the propulsion system is complex and technologically challenging, especially when there is a requirement for precise control of power and direction. Further physical scaling laws, associated with friction, heat generation and dissipation also hinder the design of micromachines, an issue which is compounded by the dominance of the fluid viscosity at low Reynolds numbers (characteristic of microflows). These issues, when combined with other constraining and pertinent issues, including biofouling and entanglement of debris, have restricted the practical application of these motors.

**Figure 1:**
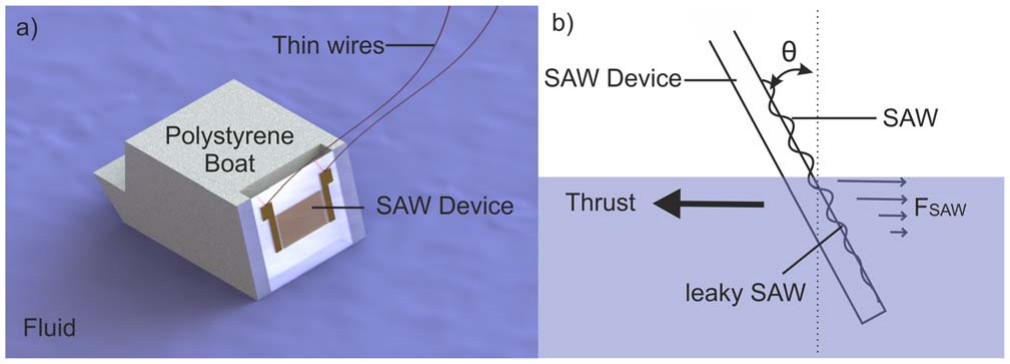
Schematic of the vessel and Physical principle. (a) Illustration of the vehicle, the SAW device is positioned at the back. (b) Schematic of the physical principle. The SAW propagates along the surface and refracts into the water producing a force (F_SAW_) and a thrust in the opposite direction.

We now show, for the first time, a SAW propulsion system with no moving parts, based upon acoustic streaming. This new, low cost SAW device differs from ultrasonic thrusters (UST) in its high efficiency in operation and its ease of fabrication [11], [12], [13]. We demonstrate the application of this technique by propelling a millimetre scale vessel illustrated in Fig. 1(a). The high frequency ultrasonic wave is attenuated in the liquid giving silent propulsion. We also demonstrate frequency dependent control of the direction of travel using a tuneable surface acoustic waves device [14]. The SAW device with its active surface, also has an intrinsic ability to reduce biofouling which would be a major advantage compare to rotary propellers [15], [16].

Our SAW micromotor is produced by standard lithography of interdigitated electrodes (IDT) on lithium niobate (LiNbO_3_), using methods commonly used throughout industry. The motor is immersed into a liquid and an acoustic wave is generated which propagates into the fluid. The acoustic waves are partially absorbed, resulting in a decrease in the mean momentum flux as the waves travel through the fluid. A net force is associated with this decrease in the momentum flux.

The equations governing acoustic streaming have already been described by Nyborg, Lighthill, Eckart, as well as others [17], [18], [19]. In our particular case, the acoustic Reynolds number (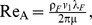 where ρ_F_, v_1_, λ_F_, μ, are respectively the fluid density, particle velocity, wavelength in the fluid and dynamic viscosity) range from 0.02 to 2, the particle velocity v_1_ from 0.01 to 1 m/s and fluid velocity from 0.1 to 10 mm/s. Re_A_ being smaller than 1 in most of the cases and the particle velocity being much higher than the fluid velocity, we make here the assumption that the streaming is “slow” and use Nyborg's equations to describe the force arising from the SAW streaming. The streaming force can be written as [15], [17], [20]:



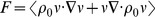
(1)Where ρ_0_ is the constant equilibrium density, ν is the oscillatory particle velocity and the brackets denote the time average.

SAW becomes a leaky SAW when in contact with a fluid as shown in Fig. 1(b). Under these conditions the SAW is refracted into the liquid with an attenuation length of the surface wave of 4 mm (F = 11.83 MHz), which is given by:



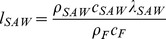
(2)Where ρ_SAW_ and ρ_F_ are the substrate and fluid densities, c_SAW_ and c_F_ and the wave velocities and λ_SAW_ is the wavelength of the SAW. The waves are refracted into the liquid as a longitudinal wave at the Rayleigh angle of 23° given by:



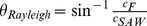
(3)To calculate the force arising from the SAW streaming, we take in account that the propagation constant k_R_ for SAW is a real number, while for leaky SAW, k_L_ is a complex number. Thus, the particle displacement (u_x_, u_z_) in the liquid can be expressed in the following form:
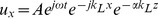
(4)

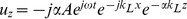
(5)Where
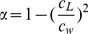
(6)a is the amplitude of the wave, ω = 2πf is the angular frequency; cL and cw are the leaky and longitudinal wave velocity.

By replacing the particle displacement of Equation 4 and 5 with the particle velocity v = δu/δt and substituting into Equation 1, the force F_x_ and F_z_ can be obtained. Since the force arising from the SAW streaming is given by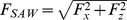, it can be expressed as:



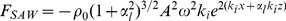
(7)Where 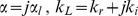


This force is directed at the Rayleigh angle and can be used to drive a vehicle in water. It has been already shown that forces arising from the SAW streaming is much greater than the force arising from a bulk wave [20].

## Results and Discussion

**Figure 2:**
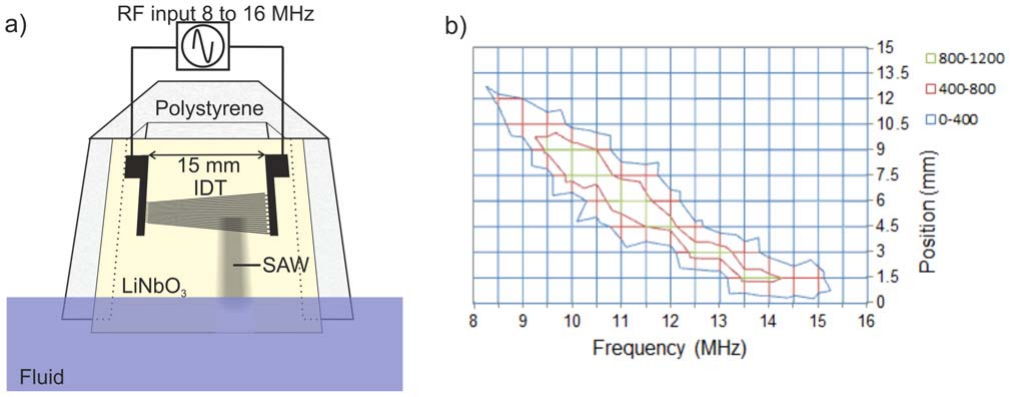
Schematic and characterization of the SAW micromotor. (a) Schematic of the vehicle (back view). A sinusoidal wave is applied to the IDT on a piezoelectric substrate to generate the surface waves, which propagate along the surface into the fluid. The SAW device is in contact with the polystyrene structure on a rail. (b) View of the surface displacement of the acoustic waves on the LiNbO_3_ wafer as a function of the input frequency and the position along the fingers for a slanted IDT, shown in Figure 2(a). The legend is the displacement in pm. The aperture of the wave beam is consistently around 3 mm. Additional sidebands expected for a tapered interdigitated transducer are not visible as their amplitudes are lower than 400 pm.

In the example shown in this paper, the vehicle consists of a floating toy made in expanded polystyrene (length  = 50 mm, width  = 35 mm, height  = 30 mm, weight  = 4.3×10^−3^ kg) as shown in Fig. 1a or a rubber duck (length  = 55 mm, width  = 50 mm, height  = 40 mm, weight  = 16.3×10^−3^ kg). The SAW device fixed at the back for propulsion. We used two different designs of IDT, including a standard IDT (Fig. 1a) and a slanted (or tapered) IDT (Fig. 2a) [14].

The SAW device was fixed to the toy with an angle of 23° with respect to the vertical in order that the waves refracting into the fluid at the Rayleigh angle of 23° propagates parallel to the surface in the fluid. The immersion of the device was 4 mm, which is the calculated length attenuation, to provide a maximal thrust efficiency as shown in Fig. 1(a–b) and 2(a).

**Figure 3:**
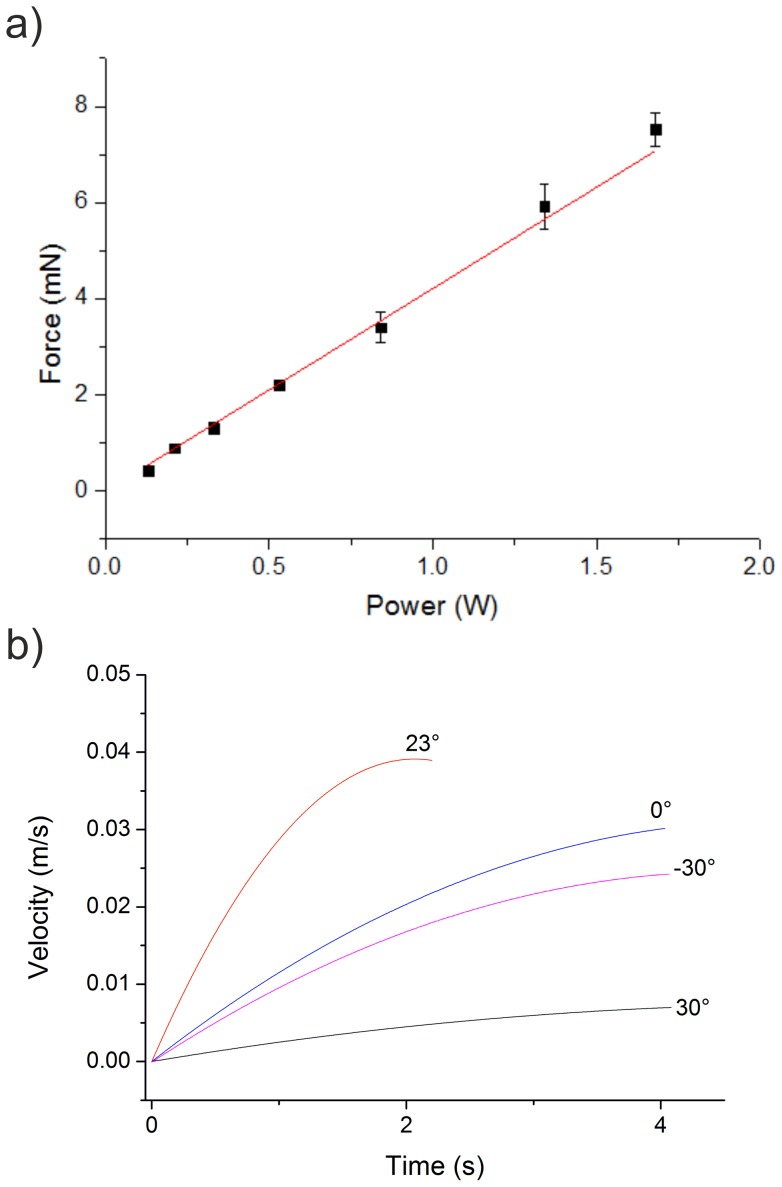
Thrust and speed. (a) Thrust force produced as a function of the acoustic power. (F = 11.83 MHz). Error bars are standard deviations (n = 5). (b) Velocity profile of the vessel during acceleration from stationary to its maximum velocity with the SAW device oriented at different angles θ (F = 11.83 MHz, power  = 1.4 W). The maximum velocity is reached by the device at 23°.

Videos were taken and analysed using ImageJ (http://rsbweb.nih.gov/ij/) to extract information concerning the velocity and the ability to control direction. The velocity profiles were further analysed to calculate the drag force and the thrust of the boat. The thrust was measured as a function of the acoustic power for the standard IDT oriented at 23° and is shown in Fig. 3. The power of the surface acoustic wave can be determined by calculating the transmission co-efficients of the IDT and the acoustic wave along its path. The standing wave ratio of the IDT at 11.83 MHz was 2.228 VSWR, leading to a mismatch loss of 0.72 dB. However, the SAW is excited in two directions on the substrate, leading to an additional reduction of the actuating power by −3 dB, since only one was transmitted into the water. Thus the acoustic power can be calculated as the electric power attenuated by 3.72 dB, since the SAW is then completely refracted into the liquid with an attenuation length of the surface wave of 4 mm according to equation 2 for a frequency of 11.83 MHz.

The force was measured by calculating the drag coefficient of the vessel and measuring the maximal speed as a function of input power. Since the thrust and the drag force are equal when the speed remains constant, by measuring the drag force we can calculate the thrust. As shown in Fig. 3a, the force is linear with the acoustic power, generating 8 mN for an input power of 1.7 W, which is 10 times more efficient than the ultrasonic thrusters [12], [13].

The orientation of the SAW device has an impact on the thrust. From the theory, it is known that the SAW refracts at the Rayleigh angle and thus the force arising from the SAW is directed at this angle. To verify this, we propelled the vessels by orienting the SAW device with different angles, θ with respect to the vertical (30°, 23°, 0° and −30°). As expected, at 23° orientation, the vessel accelerates at the highest rate, to the highest velocity (confirming that the Rayleigh angle is the most efficient coupling, notwithstanding the difference of drag force due to the variation in the design).

As stated previously, the SAW is transmitted into the fluid with an attenuation length of 4 mm, thus the immersion depth of the device above this length has an insignificant effect on the thrust. However a minimal immersion depth was preferred to reduce the drag force as much as possible. This could be achieved by increasing the frequency. We tested several immersion depths and find out that it was possible to propel the boat with depth lower than 1 mm. Due to the design of the boat, no significant differences in the maximum speed were found between depths from 1 mm to 4 mm. Above 4 mm, the maximum speed start to decrease due to the increase of the drag force.

**Figure 4:**
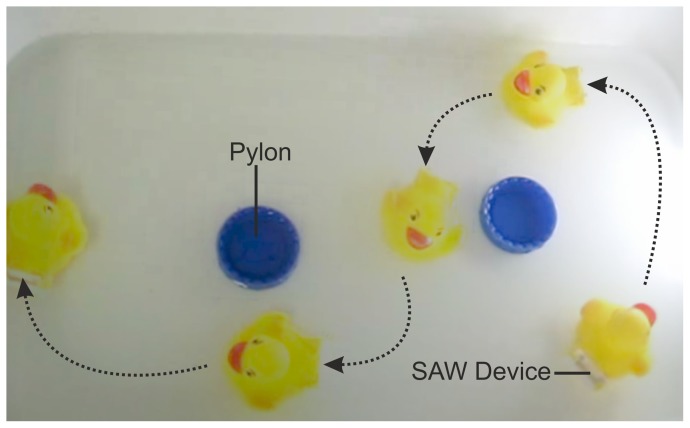
Direction control using tuneable surface acoustic waves. The image is a superposition of still from a movie every ∼3 sec. A frequency of 13.2 MHz is used to turn right and 9.2 MHz to turn left. A duck is used here for aesthetic purpose.

To illustrate the control of direction using the tuneable acoustic wave technique, the SAW device was placed in the vessel. The directional control relies on the ability of the tapered IDT to generate the wave at different positions along the aperture, with the force propagating from the SAW being generated on the side of the device and creating a torque. As showed in Fig. 4 and in Video S1 in supplementary information, both left turn and right turn are performed using respectively frequency of 9.2 MHz and 13.2 MHz. We measured the torque (τ) by first estimating the moment of inertia (I) of the rubber duck by considering it as an ellipsoid (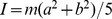) and measured the angular acceleration (α) from the videos. As 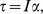 we were then able to estimate that torque in the order of 10 nNm for a frequency of 13.2 MHz and power of 1.4 W.

Finally, we investigated the heating of the device using a thermal camera. The vessel was maintained immobile in the water while images of the device were recorded with the thermal camera for several input power. The temperature of the piezoelectric increase only by 3°C on the side of the device in contact with the water for an electrical input power of 4 W, while it increased by ∼15°C on the upper side, as shown in Fig. S1 in the supplementary information. This can be explained by the fact that the energy is fully transmitted into the fluid on the lower side. To overcome the heating on the upper side, a single phase unidirectional transducer (SPUDT) could be used, which, in turn, could also increase the efficiency of the device.

Previously, it has been proposed that the single cell micro-organism cyanobacteria use waves travelling along its outer cell membrane as a surface waves, thereby providing a method of propulsion without flagella [21]. As the waves propagate along the cell membrane, they induce a streaming in the surrounding liquid and generate a force [22]. Although these waves probably move at a slower speed and have a larger z-displacement than the piezoelectric SAW, the mechanism of a propulsion system based upon generating acoustic streaming, is an interesting analogy.

In conclusion, we show for the first time a propulsion system based on SAW streaming. This technique is low cost and easy to fabricate, allowing for propulsion without any moving parts. Control of direction is inherent to the design of the device, if a slanted IDT is used. Moreover, the SAW device is scalable from millimetre size down to micrometre by scaling the electrode spacing down to 2 μm for example, and increasing the frequency to 2 GHz, using state of the art of fabrication techniques. Thus, this new technique will not only find application mostly in biomedical and aeronautic field but should also find interest in fundamental biophysical research on the propulsion of microorganisms.

## Materials and Methods

### Fabrication

The SAW device was fabricated on a 128° Y-cut X-propagating 3 inch LiNbO_3_ wafer and consisted of 10 interdigitated electrodes to form the pair of IDTs. The electrodes separation and their width was fixed at 83 µm for a standard IDT with an aperture of 10 mm and varied linearly from 62.5 µm to 125 µm along the aperture of 15 mm of the slanted IDT. The fabrication of such structures involved the LiNbO_3_ wafer being spin coated by S1818 photoresist, pattern transfer using standard photolithography and developed using Microdev, and evaporation of successively 20 nm of a titanium adhesion layer, prior to deposition of 100 nm of gold. Lift-off was then performed in acetone.

### Experimental set-up

The SAW devices were characterized using an Agilent Technologies E5071C ENA series network analyser and a vibrometer (Polytec). The SAW devices were connected to a signal generator (Agilent Technologies MXG Analog Signal Generator N5181A) in conjunction with an amplifier (Mini Circuits ZHL-5W-1, 5–500 MHz) and a 3 A, ±24 V DC power supply to generate the acoustic waves. Extra-thin wires were used to minimize their effect on the toy movement.

## Supporting Information

Figure S1 **Temperature control.** Temperature of the SAW Device after 1 minute as a function of the input electrical power.
(TIF)

Video S1 **Video of direction control.** Actuation of the vehicle using tuneable surface acoustic waves technique for the control of direction.
(AVI)
