# Supplementary figures and images for: Swimming Using Surface Acoustic Waves
Source: PLoS One. 2013 Feb 19;8(2):e42686. doi: 10.1371/journal.pone.0042686 (PMC3576398; doi:10.1371/journal.pone.0042686)

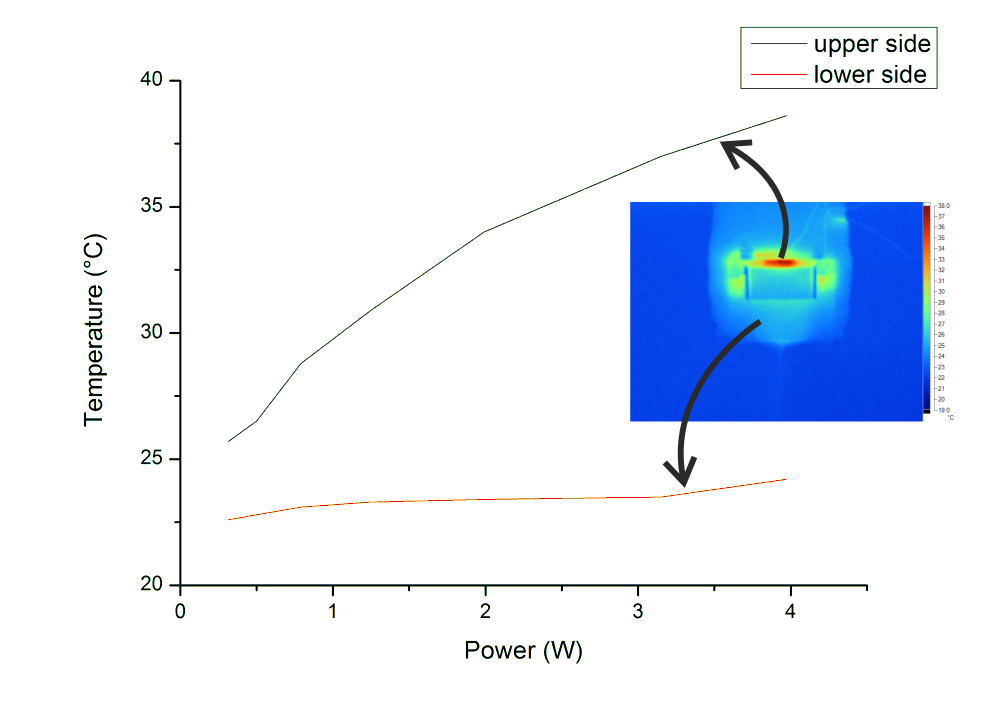

Supplement: Figure S1 — Temperature control. Temperature of the SAW Device after 1 minute as a function of the input electrical power. (TIF) [file pone.0042686.s001.tif]
